# The transport of anti-HIV drugs across blood–CNS interfaces: Summary of current knowledge and recommendations for further research

**DOI:** 10.1016/j.antiviral.2008.12.013

**Published:** 2009-05

**Authors:** Lavanya Varatharajan, Sarah A. Thomas

**Affiliations:** a St George's Hospital, Cranmer Terrace, London SW17 ORE, UK; b King's College London, Pharmaceutical Sciences Research Division, Hodgkin Building, London Bridge, London SE1 1UL, UK

**Keywords:** BBB, blood–brain barrier, P-gp, P-glycoprotein, MRP, multi-drug resistance associated protein, HAART, highly active antiretroviral therapy, PIs, protease inhibitors, NRTIs, nucleoside reverse transcriptase inhibitors, NNRTIs, non-nucleoside reverse transcriptase inhibitors, NtRTIs, nucleotide reverse transcriptase inhibitors, HAD, HIV-associated dementia, HIVE, HIV encephalitis, MND, mild neurocognitive disorder, MDR-1, multi-drug resistance gene 1, BCRP, breast cancer resistance protein, OAT, organic anion transporter, OATP, organic anion-transporting polypeptide, CSF, cerebrospinal fluid, CNS, central nervous system, AIDS, acquired immunodeficiency syndrome, HAD, HIV-associated dementia, ABC, ATP binding cassette, SLC, solute carrier superfamily, ddI, 2′,3′-dideoxyinosine, ddC, 2′3′-dideoxycytidine, 3TC, (−)-2′-deoxy-3′-thiacytidine, AZT, zidovudine, ENT, equilibrative nucleoside transporter, CNT, concentrative nucleoside transporter, ET-1, endothelin-1, LPS, lipopolysaccharide, Blood–brain barrier, Choroid plexus, HIV, HAART, Transporters, P-Glycoprotein, Antiretroviral drugs

## Abstract

The advent of highly active antiretroviral therapy (HAART), which constitutes HIV protease inhibitors, nucleoside reverse transcriptase inhibitors, non-nucleoside reverse transcriptase inhibitors and nucleotide reverse transcriptase inhibitors, has dramatically reduced the morbidity and mortality associated with human immunodeficiency virus (HIV) infection in resource-rich countries. However, this disease still kills several million people each year. Though the reason for therapeutic failure is multi-factorial, an important concern is the treatment and control of HIV within the central nervous system (CNS). Due to the restricted entry of anti-HIV drugs, the brain is thought to form a viral sanctuary site. This not only results in virological resistance, but also is often associated with the development of complications such as HIV-associated dementia. The CNS delivery of anti-HIV drugs is limited by the blood–brain and blood–CSF interfaces due to a combination of restricted paracellular movement, powerful metabolic enzymes and numerous transporters including members of the ATP binding cassette (ABC) and solute carrier (SLC) superfamilies. A better appreciation of the transporters present at the brain barriers will prove a valuable milestone in understanding the limited brain penetration of anti-HIV drugs in HIV and also aid the development of new anti-HIV drugs and drug combinations, with enhanced efficacy in the CNS. This review aims to summarise current knowledge on the transport of anti-HIV drugs across the blood–brain barrier and the choroid plexus, as well as provide recommendations for future research.

## 1 Introduction

Initially recognised in a handful of cases during the early 1980s, human immunodeficiency virus (HIV) has infected an estimated 33 million people worldwide (UNAIDS Epidemic Update, 2008), causing the acquired immunodeficiency syndrome (AIDS). In 2007 the HIV/AIDS epidemic killed approximately 2 million individuals. The central nervous system (CNS) is a major target of HIV, becoming infected in the primary stage of infection and this continues in the majority of untreated seropositive individuals throughout the course of infection.

HIV infection in the CNS is associated with the development of asymptomatic neurocognitive impairment, HIV-associated mild neurocognitive disorder (MND), and eventually HIV-associated dementia (HAD) or AIDS dementia complex that manifests as a clinical syndrome of cognitive, motor and behavioural dysfunction (Antinori et al., 2007). These neurological complications arise most likely due to the early penetration of HIV-1 into the CNS via infected immune cells such as CD4^+^ T lymphocytes, dendritic cells, monocytes and macrophages, which are all cellular reservoirs of HIV-1 (Valcour and Paul, 2006; McArthur, 2004; Blankson et al., 2002). A key feature of HIV populations in the CSF is that initially they may be identical to that in the plasma, but as the infection progresses the viral populations diverge, with the greatest divergence being observed in patients with HAD (Wong et al., 1997; Strain et al., 2005).

In the late stages of AIDS, the CNS is also vulnerable to several severe opportunistic diseases including cryptococcal meningitis and primary CNS lymphoma. All these neurological conditions were initially associated with high morbidity and mortality, and this continues to be the situation in resource-poor countries where highly active antiretroviral therapy (HAART) is not readily available (Table 1). However, where there is wide-spread use of HAART there has been a profound reduction in the incidence/severity of HAD (d’Arminio et al., 2004) and the major CNS opportunistic infections, although the prevalence of minor HIV-1 associated cognitive impairment appears to be rising. Various factors including toxicity, insurgence of drug resistance, poor medication adherence and sometimes limited access to HAART, contribute to this phenomenon. It has also been speculated that the constant presence of HIV and the associated immunoactivation and inflammation in CSF is linked to this more subtle form of brain injury. As elevated viral loads in the CSF, predict subsequent neuro-cognitive impairments (Ellis et al., 2002). Overall it would suggest that treatment of CNS disease associated with HIV-infection may be sub-optimal and clinical data has suggested that the CNS effectiveness of anti-HIV drug regimens can be significantly improved by treating with CSF-penetrating antiretroviral drugs in patients with cognitive impairments (Letendre et al., 2004, 2008a,b).

The classification of the antiretroviral drugs into either low-CNS penetration (rank 0), intermediate CNS penetration (rank 0.5) or high-CNS penetration (rank 1) was based on extensive literature reviews and considered the physico-chemical characteristics of the drug, measured CSF concentrations and effectiveness in the CNS (Table 2) (Letendre et al., 2008a). Drug combinations are ranked by combining the individual drug rankings. However, this simple ranking for drug combinations does not allow for other drug properties, such as drug interactions at the blood–CNS interfaces to be considered, and these factors may actually contribute to lack of, or in fact enhanced, CNS efficacy of specific drug combinations. Recent progress strongly suggests that certain transporters in the BBB impede the access of anti-HIVs to the CNS and that pharmacological modulation of these transporters could be used to our advantage (Eilers et al., 2008).

In order to examine drug interactions at the transporter level of the blood–CNS interfaces in more detail, a clear picture of drug movement into the CNS is clearly warranted. This may prove useful in fine-tuning this ranking scheme and is the focus of this review. We will start with a summary of current anti-HIV drugs and how molecules are restricted from crossing the blood–CNS interfaces. This will then be followed by a brief overview of those membrane transporters considered to be relevant to the disposition and effectiveness of nucleoside reverse transcriptase inhibitors (NRTIs), non-nucleoside reverse transcriptase inhibitors (NNRTIs), nucleotide reverse transcriptase inhibitors (NtRTIs) and protease inhibitors (PIs). Possible future research directions will then be discussed.

## 2 Anti-HIV drugs

According to US and British guidelines, the goal of HIV therapy must always be to achieve a viral load of <50 copies/mL within 4–6 months of starting treatment (Gazzard, 2008; Department of Health and Human Services, 2008). Traditionally, the main classes of anti-HIV drugs used include NRTIs, NNRTIs, NtRTIs and PIs (Table 1). However, other classes are also available including integrase inhibitors and entry inhibitors such as fusion and CCR5 inhibitors (Qian et al., 2009). HAART comprises the combined use of three or more anti-HIV drugs. The current recommended first line of treatment is a NNRTI, efavirenz, with the nucleoside backbone Truvada^®^ (emtricitabine and tenofovir disoproxil fumarate) and Kivexa^®^ (abacavir and lamivudine) (Gazzard, 2008).

## 3 Blood–brain and blood–CSF barriers

**Fig. 1:**
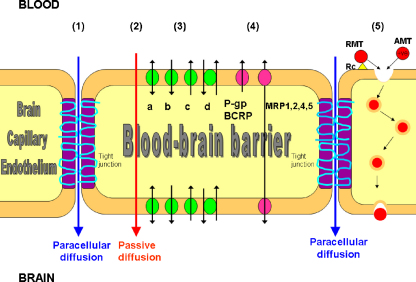
Permeation mechanisms across the BBB: (1) paracellular diffusion between the cells of the capillary endothelium. (2) Transcellular diffusion; solutes with sufficient lipid solubility may passively diffuse through the cell membranes of the endothelial cells and enter the brain. (3) Carrier-mediated transport of more hydrophilic molecules is via SLCs inserted into the luminal and/or abluminal membrane. These may be (a) facilitated bi-directional carriers operating in the direction of the concentration gradient, (b/c) uni-directional into or out of the cell (d) co-transporters/exchangers co-transporting or exchanging another solute or ion in the same or opposite direction. (4) Active efflux carriers (ABC transporters) may pump out a wide range of passively penetrating solutes either from the cytoplasmic compartment or directly from the cell membrane. (5) Transcytosis can also occur which may be non-specific (fluid phase, very limited at the BBB) or specific. Specific mechanisms include absorptive-mediated transcytosis (AMT), which involves an electrostatic interaction between a positively charged substance and the negatively charge plasma membrane surface, or receptor-mediated transcytosis (RMT), which involves a receptor (Rc) and is highly selective for specific molecules.

Substances can cross non-barrier capillary walls by a variety of routes, either between the cells of the endothelium (paracellular movement), directly through the cell wall (active transport, facilitated and/or passive diffusion) or through vesicular transport (endocytosis). However, the cerebral capillaries are the site of the blood–brain barrier (BBB) and consequently these three routes are restricted by the presence of tight junctions between the endothelial cells, the lack of intracellular fenestrations in brain endothelial cells, the paucity of endocytotic vesicles as well as by the presence of multiple metabolic enzymes and diverse transport systems (Fig. 1). Thus the entry of anti-HIV drugs/substances by unregulated pathways or by leakage is limited.

In addition, the choroid plexus acts as a barrier between the circulating blood and cerebrospinal fluid (CSF) and is also the site of production of the latter. They are located in the lateral, third and fourth ventricles and their barrier function is attributed to the tight junctions between the epithelial cells, in addition to the metabolism and efflux that occurs within the tissue itself (Strazielle et al., 2004). However, the molecular composition of the intercellular junctional complexes and the transporters present at the BBB (Gazzin et al., 2008) differ from that on the blood–CSF barrier, which means that drug movement into the brain varies depending on its main route of entry. Consequently, although the CSF is an accessible body fluid that provides insight into the brain infection and drug penetration, it can also diverge in important ways from the brain itself. An understanding of anti-HIV drug movement across both the BBB and the blood–CSF barrier is therefore of special interest.

## 4 Transporters

A large spectrum of transporters exist for different substrates and following the sequencing of the human genome it is believed that approximately 500–1200 genes encode drug transporters (Sakaeda et al., 2004). These carriers have been classified into two main groups, designated the ATP-binding cassette (ABC) transporters and the solute-carrier (SLC) superfamily (Loscher and Potschka, 2005).

### 4.1 ABC transporters

ABC transporters are the most extensively studied group, which are responsible for the cellular extrusion of a variety of molecules by using energy produced from ATP hydrolysis (Loscher and Potschka, 2005). P-Glycoprotein (P-gp) (also known as MDR-1 or ABCB1) is the most characterised ABC transporter. Other members of the superfamily include multi-drug resistance-associated proteins (MRPs) (also referred to as ABCC transporters) and breast cancer resistance protein (BCRP) (also known as ABCG2). Recent studies have demonstrated that the expression of these transporters at the BBB and on other cells such as lymphocytes, CD4^+^ T cells and microglia, play a crucial role in permitting the entry of anti-HIV drugs into cellular and anatomical reservoirs of HIV-1.

#### 4.1.1 P-gp

P-gp, first discovered in 1976, is a 150–180 kDa membrane protein encoded by the multi-drug resistance gene 1 (MDR-1). It is widely expressed in tissues such as the liver, kidney and intestine, as well as the luminal membrane of the BBB (Cordon-Cardo et al., 1989; Jette et al., 1993, 1995; Soontornmalai et al., 2006) and the choroid plexus epithelium (Rao et al., 1999; Gazzin et al., 2008). At the BBB it appears to be important in protecting the brain from hydrophobic molecules and drugs. However, its function at the blood–CSF barrier is ambiguous and it may not have a major neuroprotective role at this site (Gazzin et al., 2008). Uncharged or weakly basic molecules are most efficiently transported by P-gp, but acidic compounds can also be transported. A large body of evidence suggests that PIs are substrates of P-gp and as a result the limited ability of these drugs to transverse the blood–brain barrier is attributed to the activity of this efflux transporter (Kim et al., 1998; Polli et al., 1999; Choo et al., 2000; Park and Sinko, 2005; Bachmeier et al., 2005; Eilers et al., 2008).

In comparison to PIs, the interaction of P-gp and NRTIs has been less extensively studied. Recently a study provided *in vivo* and *in vitro* evidence that the nucleoside reverse transcriptase inhibitor, abacavir ([(−)-(1*S*, 4*R*)-4-[2-amino-6 (cyclopropylamino)-H-purin-9-yl]-2-cyclopentene-1-methanol; 1592U89]) is a P-gp substrate (Shaik et al., 2007; Giri et al., 2008). In fact it is likely that P-gp is the dominant transporter limiting the CNS penetration of abacavir (Giri et al., 2008). Similarly, earlier studies showed that both HIV-infected T cell and monocytic cell lines had increased P-gp expression which accumulated significantly less zidovudine (azidodeoxythymidine (AZT)) in comparison to uninfected cells (Gollapudi and Gupta, 1990). Likewise, a decrease in AZT accumulation in P-gp-over-expressing CEM VBL100 cells with a corresponding decline in antiviral efficacy of the drug has been observed (Antonelli et al., 1992).

Using P-gp in Caco-2 cell lines, the NNRTIs nevirapine, efavirenz, and delavirdine were found not to be substrates of this transporter. However, all of these drugs were found to induce the expression and function of P-gp, with nevirapine being the more potent inducer compared with the other two NNRTIs (Stormer et al., 2002). Alternatively, a recent study demonstrated that the effect of P-gp on intracellular HIV-1 replication may be more clinically relevant than the efflux function of P-gp on PIs. The data suggested that high-cellular P-gp activity corresponds with a lower intracellular HIV-1 load *in vivo* (Sankatsing et al., 2007). Interestingly, Langford et al. (2004) showed that AIDS patients with HIV encephalitis (HIVE) have higher brain P-gp levels than HIVE-negative patients. However, despite studies showing an upregulation of P-gp in HIV-1 infected macrophages, CD4^+^ T lymphocytes and glial cells (Langford et al., 2004), the pump function of P-gp in HIV-1 infected patients is thought to be decreased (Sankatsing et al., 2004).

Recent experiments using primary culture of rat astrocytes have demonstrated that both the expression and the transport function of P-gp are downregulated following exposure to HIV viral envelope protein, gp120. Collectively, these crucial glial cells that harbour the virus within the CNS are thought to form a dynamic barrier behind the BBB to further impede the access of anti-HIV drugs to sites of infection within the CNS (Ronaldson and Bendayan, 2006). Furthermore, using intact, isolated rat brain capillaries, Hartz et al. (2004) revealed that subnanomolar to nanomolar concentrations of the hormone endothelin-1 (ET-1) rapidly and reversibly attenuated P-gp-mediated transport function over the short term (minutes). This effect was found to be due to the stimulation of the ET_B_ receptor with subsequent activation of nitric oxide synthase and protein kinase C. The release of ET-1 has been apparent in a number of CNS disorders including HIVE (Hartz et al., 2004) and AIDS dementia complex however the effect of ET-1 on brain capillary permeability remains controversial, with some studies claiming that ET-1 significantly increases brain permeability and others suggesting no effect. This discrepancy can be attributed to the different durations of the experiments. An increase in permeability was observed over hours to days, raising the possibility that capillary permeability may remain unchanged during early ET-1 exposure (Hartz et al., 2004).

Inflammation is a central pathophysiological mechanism in the majority of CNS diseases and is reproduced experimentally by the injection of the bacterial endotoxin—lipopolysaccharide (LPS). Altered P-gp expression and corresponding changes in the disposition of several xenobiotics have been observed in the LPS model (Miller et al., 2008). Recent studies have demonstrated evidence in line with these findings. P-gp was downregulated via an unknown mechanism following the administration of LPS into rat intracranial ventricles. This subsequently caused an accumulation of the P-gp substrate, digoxin, within the brain (Goralski et al., 2003). Other studies have shown that the proinflammatory cytokine TNF-α causes a rapid and reversible loss of P-gp activity in rat brain capillaries.

The proposed mechanism suggested that short-term exposure to the cytokine caused TNF receptor 1 stimulation resulting in ET-1 release and consequent ETB receptor, nitric oxide synthase and protein kinase C activation. This pathway was thought to be activated by LPS to reduce P-gp transporter activity (Hartz et al., 2006). More recently, the same research group found that this initial rapid decrease in transport preceded a 2–3-h plateau at this reduced level of transporter activity, and was then followed by a rapid increase in both transporter activity and protein expression. Collectively, these findings demonstrate that chronic inflammation can tighten the BBB to CNS drugs which are P-gp substrates by upregulating P-gp expression (Bauer et al., 2007). An upregulation of P-gp in rat brain endothelium was also observed in an inflammatory pain model causing a decrease in the penetration of the P-gp substrate, morphine and consequent antinociception (Seelbach et al., 2007).

HIV-Tat, a protein thought to be responsible for the vascular abnormalities and neurotoxicity in HIV, also induces the expression of P-gp in brain endothelial cells which correlated with a functional upregulation of the transporter function of P-gp (Hayashi et al., 2005). A similar change in P-gp expression has been observed following chronic exposure of bovine brain microvessel endothelial cells to ritonavir. In fact, the HIV PI increased P-gp activity and expression in a concentration-dependent manner in this *in vitro* model of the BBB, raising the possibility that HAART could itself contribute to the brain as a HIV sanctuary site by the induction of drug transporters (Perloff et al., 2007). Collectively, these studies suggest that the selective inhibition of P-gp may facilitate the entry of PIs and certain NRTIs into viral sanctuaries and enhance the concentration of anti-HIV drugs in these sites to therapeutic levels.

#### 4.1.2 MRPs

MRPs are ATP-driven efflux transporters. Those localised at the luminal membrane of the brain capillary endothelial cells (i.e. Mrp5) contribute to the non-permissive nature of the BBB (Dallas et al., 2006). However, evidence also exists for an abluminal (Mrp1) and possibly cytoplasmic (Mrp2) expression of specific isoforms (Soontornmalai et al., 2006). Currently, direct evidence exists only for the expression of MRP-1, MRP-2, MRP-4 and MRP-5 at the BBB (Dallas et al., 2006; Soontornmalai et al., 2006; Eilers et al., 2008) and of Mrp1 and Mrp3 (but not Mrp5) at the choroid plexus (Soontornmalai et al., 2006). Interestingly the choroid plexus, but not the BBB, is the main site of blood-facing, Mrp1-dependent cellular efflux from the rat brain (Gazzin et al., 2008).

MRPs are known to co-transport drugs with glucuronide, glutathione or sulphate. Amongst the nine members of this transporter family (MRP1–9), the first five (MRP-1, MRP-2, MRP-3, MRP-4 and MRP-5) are frequently associated with the efflux of therapeutic agents. MRP-1, MRP-2 and MRP-3, transport hydrophilic anionic compounds, large molecules and peptidomimetics (Dallas et al., 2006); however, both MRP-4 and MRP-5 transport small polar compounds such as nucleosides, cyclic nucleotides and nucleoside analogs (Schuetz et al., 1999; Wijnholds et al., 2000).

The HIV PIs saquinavir, ritonavir and lopanivir were found to be substrates of MRP-1 and MRP-2 and therefore these transporters may also contribute to the limited penetration of PIs into the brain (Bachmeier et al., 2005; Park and Sinko, 2005; Janneh et al., 2005, 2007; Eilers et al., 2008). A recent *in vitro* study demonstrated that MRP-1, MRP-2 and MRP-3 were also inhibited in a concentration-dependent manner by NNRTIs (delavirdine, efavirenz and nevirapine), NRTI (abacavir, emtricitabine and lamivudine) and the NtRTI, tenofovir. This inhibition was particularly apparent for delaviridine, efavirenz and emtricitabine (Weiss et al., 2007). In addition, MRP-4 and MRP-5 are thought to be low-affinity transporters of nucleoside-based antiretrovirals (Schuetz et al., 1999; Wijnholds et al., 2000) and both have been implicated in the rapid removal of zidovudine from human brain microvessel endothelial cells (Eilers et al., 2008). Previous studies have also suggested an association between MRP-4 over-expression and increased efflux of nucleoside-based anti-HIV drugs (Schuetz et al., 1999). A previous *in vitro* study revealed that ritonavir induces the expression of MRP-1 in a concentration-dependent manner, however this was noted in human colon in adenocarcinoma cells (Gimenez et al., 2004). MRP-1 is also thought to promote HIV replication (Speck et al., 2002).

#### 4.1.3 BCRP

BCRP is a novel ABC-transporter which is localised in various tissues including cerebral endothelial cells (Eisenblatter et al., 2003). It is believed to have a similar tissue localisation to P-gp (Fig. 1). PIs such as ritonavir, saquinavir and nelfinavir have found to be effective inhibitors, but not substrates of this transporter (Gupta et al., 2004). Studies have also found that BCRP is a cellular factor involved in the resistance to NRTIs. For example, the accumulation and anti-HIV activity of AZT was significantly reduced by diminishing its metabolites, in cells overexpressing BCRP and this was reversed by fumitremorgin C, a BCRP inhibitor, suggesting that AZT is a substrate of BCRP (Wang and Baba, 2005; Wang et al., 2004).

More recently, a murine homolog of human BCRP was used to investigate the contribution of BCRP in the directional transport of abacavir and AZT. Their data provided evidence suggesting that both these drugs are substrates for Bcrp1 and further directional transport studies confirmed the role of Bcrp in the polarised transport of both abacavir and AZT *in vitro* (Pan et al., 2007). However, *in vivo* results showed that deletion of Bcrp1 has little influence on the brain penetration or overall disposition of AZT and only a moderate effect on abacavir (Giri et al., 2008). Overall, findings from these studies suggest that BCRP could contribute to drug–drug interactions observed *in vivo* following HAART. The role of this efflux protein during co-administration of anti-HIV drugs that are either substrates, inhibitors or both of BCRP, could be very valuable for the delivery of HAART to viral sanctuary sites.

### 4.2 The SLC superfamily

A large body of evidence demonstrates that the solute carrier (SLC) superfamily plays a crucial role in the efflux transport of organic compounds, organic anions particularly, across the BBB (Kusuhara and Sugiyama, 2005). Some members of this superfamily include organic anion-transporting polypeptide (OATPs), organic anion transporters (OATs) and organic cation transporters (OCTs). These ATP and sodium-independent polypeptides are expressed in a variety of tissues including the brain capillary endothelium and choroid plexus epithelial cells and regulate the movement of drugs through the brain barriers.

#### 4.2.1 OATPs

All Oatp/OATPS are members of the SLC21 family and 36 Oatps/OATPS have so far been identified in humans, rats and mice (Hagenbuch and Meier, 2004). Several studies have shown that these transporters have a role in the efflux of organic compounds. The expression of OATP1A2 and OATP1C1 have been found in the BBB and the brain, respectively. In rats, Oatp1a4, Oatp1a5 and Oatp1c1 are expressed at the BBB and the blood–CSF barrier (Kusuhara and Sugiyama, 2005). OATP has been implicated in the removal of 2′3′-dideoxycytidine (ddC; zalcitabine) from the brain and CSF (Gibbs and Thomas, 2002). Oatp2 is found at the cerebral capillary endothelium and the choroid plexus epithelium, suggesting a role for these carriers in the transport of drugs between the blood and CNS compartments (Gao et al., 1999). An Oatp-2 like transporter has been implicated in the uptake of the NRTIs, 2′,3′-dideoxyinosine (ddI, didanosine) and (−)-2′-deoxy-3′-thiacytidine (3TC, lamivudine) into the choroid plexus (Gibbs and Thomas, 2002; Gibbs et al., 2003a,b). HIV may enter the CNS via the choroid plexus and therefore transporters present at this site could play an important role in CNS efficacy of certain drugs.

#### 4.2.2 OATs

This family of transporters comprise OAT1, OAT2, OAT3, OAT4 and RST. UST1, UST3 and OAT5 are also considered as transporters of organic anions, however this has not yet been proven (Anzai et al., 2006). The recently identified urate transporter, URAT1 is very similar to RST in terms of amino acid sequence and tissue distribution. Additionally, URAT1 appears to be the human ortholog of murine RST and rat OAT1 (Eraly et al., 2004). Many drugs exist as organic anions at physiological pH levels and therefore OATs present must have a pivotal role in handling these compounds. Studies using *Xenopus laevis* oocytes showed that antiretroviral nucleoside drugs are substrates for OAT proteins despite the fact that they are not conventional organic anions (Strazielle et al., 2003).

Oat3 is present in the rat brain capillaries (Ohtsuki et al., 2002). There is no direct evidence for the expression of Oat1 at the BBB, however, it is suggested that the observed efflux of certain substances known to be Oat1 substrates from brain to blood indicates that the presence of Oat1 is likely (Sun et al., 2003). ddC and AZT are thought to be removed from the brain by a member of the OAT family (Takasawa et al., 1997; Gibbs and Thomas, 2002; Strazielle et al., 2003). OAT1 and 3 are expressed in the human, murine and rat choroid plexus (Sweet et al., 2002; Alebouyeh et al., 2003) and seem to be largely important in determining the availability of organic anions in the CSF (Eraly et al., 2004). These isoforms are likely candidates for the uptake of CSF-borne AZT and ddC (Gibbs and Thomas, 2002; Strazielle et al., 2003). However, the efflux of AZT was inhibited by specific inhibitors of both of these transporters, which makes it difficult to attribute a particular isoform to this effect.

Anthonypillai et al. (2006) investigated the distribution of a prodrug of the NRTI tenofovir, known as PMPA to the brain, CSF and choroid plexus. Interestingly, the entry of this pro-drug into the brain was negligible, but it could reach the CSF. The presence of a transporter at the level of the choroid plexus was indicated. Since hOAT1 and hOAT3 are high- and low-affinity transporters of PMPA, respectively (Cihlar et al., 2001; Izzedine et al., 2005), the involvement of these carriers was speculated however no detectable interaction was observed (Anthonypillai et al., 2006).

#### 4.2.3 OCTs

The literature concerning OCT expression in the brain is at times contradictory, and this maybe the result of variation between species and also differing area and levels of expression of the transporters within the brain (Sweet et al., 2001). A study by Slitt et al. (2002) found that mRNA for all five organic cation transporters could be detected in the rat brain. Although this work did not evaluate whether the transporters were present at the brain barriers, additional studies have provided some evidence for the presence for individual organic cation transporters at the BBB and choroid plexus. It has been established that OCTN2 is found on the luminal face (and possibly the abluminal face too) of the cerebral capillary endothelial cells of humans, rats, pigs and cows (Kido et al., 2001). OCT2, OCT3, OCTN1 and OCTN2 (but not OCT1) are also expressed in the rat choroid plexus (Sweet et al., 2001). Human OCTs play a crucial role in the initial step involved in hepatic or renal excretion of many cationic compounds. Furthermore, previous studies have demonstrated the involvement of rat OCTs and an undefined organic cation-proton exchanger in the cellular uptake of AZT and 3TC, raising the possibility that human OCTs could also be involved in the uptake of anti-HIV drugs (Minuesa et al., 2008). Moreover, Minuesa et al. (2008) revealed that hOCTs were heterogeneously expressed in primary lymphocytes, monocytes, macrophages and dendritic cells. These transporters were also upregulated upon lymphocyte activation.

A recent study suggested that the PIs, nelfinavir, ritonavir, saquinavir and indinavir are possibly inhibitors of OCT1 and OCT2 (Jung et al., 2008). This supported evidence provided by Zhang et al. (2000) that some HIV PIs may be potent inhibitors of cationic drug uptake, but poor substrates for human OCT1 (Zhang et al., 2000). Furthermore, the NRTIs, lamivudine and zalcitabine are substrates of OCT1 and OCT2 (Jung et al., 2008). Moreover, when assessing the expression of OCTs in lymph nodes of HIV-infected patients in comparison to healthy controls, Jung and colleagues found that OCT1 and OCT2 were upregulated in the lymph nodes of HIV-infected patients, indicating that the accumulation of OCT substrates would be much higher in the lymph nodes of these patients. This could be due to the activation of the immune system and effects of cytokines induced by HIV infection (Jung et al., 2008). This study implies that a sound knowledge of OCTs is crucial in understanding both drug interactions that may occur with HAART and also the role they play in regulating drug transport.

### 4.3 Nucleoside transporters

There are two main types of nucleoside transporters, classified as low-affinity equilibrative (SLC29) and high-affinity concentrative (SLC28), which are known to transport certain NRTIs.

#### 4.3.1 Equilibrative nucleoside transporters (ENTs)

There are four members of the SLC29 family. hENT1, the first characterised family member, shares similar substrate specificities with hENT2. These transporters play a pivotal role in the uptake of nucleoside and nucleobases across a membrane down their concentration gradient. Based on their sensitivity to nitrobenzythioinosine, ENTs are subdivided into equilibrative sensitive (*es*) and equilibrative insensitive (*ei*) (Baldwin et al., 2004; Thomas, 2004).

ENT1 protein, which encodes the *es* nucleoside transporter, is present on the human BBB. It is thought that this carrier is responsible for the transport of ddI across the guinea pig BBB. This was confirmed when the *es* transporter substrates, adenosine and thymidine, inhibited the brain uptake of [^3^H]ddI (Gibbs et al., 2003a). Interestingly, the *es* transporter is also active in human lymphocytes, raising the possibility that it could facilitate the transport of NRTIs into this HIV reservoir (Chan et al., 1993). ENT2, the *ei* transporter, transports purine and pyrimidine nucleosides but with a lower affinity that ENT1. However unlike ENT1, ENT2 is a carrier of AZT and can transport ddI and ddC much more efficiently than ENT1 (Baldwin et al., 2004).

Recently, Minuesa et al. (2008) found that human ENTs were the most highly expressed transporters in peripheral blood mononuclear cells and CD4^+^ T-cells and that the expression and activity of this transporter were increased by 100- and 30-fold, respectively, following stimulation of primary T lymphocytes. Determining the isoform responsible for anti-HIV drug transport across cell membranes would prove valuable in targeting these drugs to infected cells and in understanding drug–drug interactions and therapy failure (Minuesa et al., 2008).

#### 4.3.2 Concentrative nucleoside transporters (CNTs)

There are three subtypes of sodium-dependent CNTs (CNT1–3) in the SLC28 family. CNT1 is a pyrimidine specific transporter whereas CNT2 is a purine-preferring transporter which also has the ability to transport uridine. CNT3 is a broad selective nucleoside transporter (Gray et al., 2004). These transporters have been identified in the liver, intestine, kidney and choroid plexus. Redzic et al. (2005) demonstrated the presence of rENT1, rENT2 and rCNT2 mRNA and protein in primary cultures of rat brain endothelial cells and rat choroid plexus epithelial cells. rCNT3 was present at the transcript level in choroid plexus epithelium. Furthermore, they showed that the concentrative transport of nucleosides was associated exclusively with the membrane that faces brain fluids *in situ*, suggesting a role of these barriers in extruding nucleosides from the brain (Redzic et al., 2005).

Using rat and human tissues, CNT and ENT cDNAs were cloned and their functional characterisation was determined using *Xenopus* oocytes. This study revealed that CNT1, CNT3 and ENT2 but not CNT2 or ENT1 transported AZT but with a much lower affinity than endogenous nucleosides. In addition, the influx of AZT into the CSF through choroid epithelial cells was found to be unaffected by endogenous nucleosides (Yao et al., 2001; Strazielle et al., 2003). CNT has also been implicated in the transport of ddI across the BBB (Li et al., 2001). However, the role of human CNT1 as a uptake transporter for ddI and stavudine (d4T) has remained controversial, as some researchers have described such a function for hCNT1 whereas others (Minuesa et al., 2008; Chishty et al., 2004; Chang et al., 2004; Cano-Soldado et al., 2004) have argued that hCNT1/2 (and even hENT1) have a low affinity for these drugs due to the absence of the 3′OH group in the ribose ring of the structure of nucleoside analogs. Conversely, hCNT3 appears to be an efficient transporter of AZT, ddC and ddI (Minuesa et al., 2008).

## 5 Other aspects

BBB disruption during HIV infection is thought to provide a primary portal for infected cells to enter the brain and to contribute significantly to neuropathology such as HIVE and HAD (Toborek et al., 2005; Wang et al., 2008). Several processes have been attributed to the disruption of the BBB in HIV infection, in addition to those discussed earlier. Morphological changes such as an increase in the diameter of cortical vessels as well as alterations such as the thinning of basal lamina and loss of glycoproteins present in endothelial cells are all features of the disrupted BBB observed in HIV-infected patients. Furthermore, apoptosis of endothelial cells and tight junction disruption are thought to be other mechanisms by which the integrity of the BBB is impaired in HIV (Toborek et al., 2005).

HIV infection is also associated with a wide range of vasculitides including that affecting small, medium and large vessels and one common feature amongst all vasculitides seen in HIV infection is the underlying inflammation of the vessel wall (Guillevin, 2008). A recent study has also indicated that HIV-1 increases the sensitivity of the BBB to disruption by systemic lipopolysaccharide, which is elevated in HIV-1-infected individuals (Wang et al., 2008). Furthermore, monocytes that were infected with HIV-1 provirus were more able to cross the BBB particularly after lipopolysaccharide administration. Interestingly, HAART has been shown to reduce the elevated systemic levels of lipopolysaccharide present in HIV-1-infected individuals (Brenchley et al., 2006). It has thus been suggested that HAART may reduce the incidence of HAD by not only reducing HIV-1 replication but also by reducing circulating lipopolysaccharide and thereby reducing compromise of the BBB and migration of HIV-1-infected monocytes from the systemic circulation into the brain (Wang et al., 2008). However, it may also be that disruption of the brain barriers facilitates the entry of drugs into the CNS and consequently alleviates the symptoms of HAD. The potential toxic effects this may have due to excessive drug concentrations accessing the brain (see below) or other unknown effects precipitated by higher concentrations of anti-HIV drugs in the CNS are important factors that remain to be explored (Toborek et al., 2005; Gimenez et al., 2004).

Both *in vitro* and *in vivo* studies, as well as clinical observations, have shown that conventional anti-HIV drugs can have significant toxic effects. HAART has been directly associated with the development of endothelial dysfunction and consequently with cardiovascular diseases. Clinical toxicities of NRTIs include peripheral neuropathy, skeletal myopathy, lactic acidosis and hyperlactatemia (Kline and Sutliff, 2008). There are two types of CNS adverse reactions associated with HAART (1) direct drug toxicity and (2) immunopathology related to immune restoration and toxicity (Price and Spudich, 2008). Although there is little evidence to suggest that NRTIs and PIs have direct adverse effects on the CNS, the NNRTI efavirenz clearly affects CNS function, and its use is complicated by neuropsychiatric symptoms such as dysphoric dreams. Additionally, one report suggested an interactive effect of efavirenz and tenofovir on neuropsychiatric symptoms, which were however, fully reversible following the cessation of treatment (Price and Spudich, 2008). Furthermore, a recent study has revealed that prolonged HAART usage and aging may play a role in the overall increase in amyloid deposition in HIV dementia mediated by either inhibition of insulin degradation enzyme or disrupted amyloid precursor protein transport (Nath and Sacktor, 2006).

Recently the term “Immune reconstitution inflammatory syndrome” (IRIS) has been created to recognise the improvement of the immune system generally seen a few weeks after the initiation of HAART. Paradoxically, IRIS also results in a decline in clinical status and is predominantly associated with a huge rise in the CD4 T-lymphocyte count as well as a reduction in the peripheral HIV RNA viral load. If IRIS is untreated and affects the CNS, then it is associated with significant morbidity and mortality. Recently, IRIS fatally affected two patients with HIV dementia, despite control of the virus in the periphery (Nath and Sacktor, 2006).

## 6 Conclusion and future directions

The HIV/AIDS pandemic has lasted almost 3 decades, and a wide range of anti-HIV drugs have been developed in an attempt to ameliorate the infection and ultimately eliminate this highly fatal disease. To tackle HIV and prevent the formation of viral sanctuary sites, antiretroviral drugs must be able to access the brain. However the normal function of the BBB and blood–CSF barrier to shield the brain from harmful substances and provide a precisely regulated unique environment in the CNS, hinders the penetration of anti-HIV drugs into the brain, promoting viral replication, the development of drug resistance and ultimately sub-therapeutic concentrations of drugs reaching the brain, leading to therapeutic failure. Consequently, Letendre's CNS penetration-effectiveness ranking concept for quantifying antiretroviral drug penetration into the CNS is clearly worthy of serious consideration as an additional tool in designing more effective drug treatment strategies (Letendre et al., 2008a). However, it is important that the actual raw information that was used to determine each drug's individual rank becomes available, so that the ranking scheme could be further and more easily enhanced by considering drug–drug interactions at the level of blood–CNS interfaces and possibly other factors (e.g. toxicity issues) as they come to light.

The plethora of transporters expressed at the brain barriers all act as selective gatekeepers, and this remains a major obstacle for antiviral therapy. Multi-drug transporters have overlapping substrate specificity for numerous hydrophobic compounds and there seems to be considerable overlap in the efflux transporters used by certain anti-HIV drugs raising the possibility that inhibiting more than one transporter at a time may be one approach to improve CNS delivery of anti-HIV drugs. Although certain studies have demonstrated that simultaneously inhibiting multiple transporters can augment the concentration of anti-HIV drugs in the CNS, this would not appear to be a viable strategy, because it leaves the brain susceptible to damage by putative toxins. However, pharmacological modulation of individual transporters by concomitantly administering HAART with transporter-specific inhibitors may be a more successful alternative to enhance anti-HIV drug levels in the brain, without causing these general and systemic adverse effects (Eilers et al., 2008).

Importantly, the relative functional importance of specific transporters in individual tissues is slowly being revealed. In a recent study by Kamiie et al. (2008) the development of a sensitive and simultaneous quantification method using *in silico* peptide selection criteria and multi-channel multiple reaction monitoring was implemented. By using this novel method, they were able to determine simultaneously the absolute protein expression levels of multiple membrane transporters in mouse brain capillary endothelial cells. Consequently, a quantitative atlas of membrane transporter proteins was constructed. Of relevance to this review, the transporters quantified in mouse BBB were Mdr1a, Mrp4, Bcrp, Oat3 and Oatp2 (Kamiie et al., 2008). Their findings regarding protein expression levels were consistent with previous functional data (Schinkel et al., 1997). This advance in pharmacoproteomics and its extension to human tissues will not only provide significant insight into transport proteins at the BBB and other tissues, but will also hasten the translation of preclinical work to clinical studies and drug development (Kamiie et al., 2008).

Information regarding the interaction of anti-HIV drugs with recently discovered novel transporters such as URAT1 and RST are also needed, in order to acquire a complete picture of the impediments that restrict anti-HIV drug transport across the brain barriers. Conversely, the interaction between newer drugs, such as Enfuvirtide and Maraviroc, and transporters also should be fully investigated. Additionally, it would be valuable to explore whether those transporters that have altered expression during HIV infection affect the penetration of HAART across the brain barriers, and if so, whether this can be pharmacologically modulated. Modulating transporters is one method by which drug delivery into the brain could be improved (Miller et al., 2008). Thus rather than modifying substances in order to allow them to penetrate the BBB, a reasonable alternative strategy would be to modify the permeability of the BBB. Recent studies have explored factors responsible for the modulation of P-glycoprotein, Mrp2 and BCRP and the signalling mechanisms responsible for this (Bauer et al., 2004; Imai et al., 2005; Wang et al., 2006). Furthermore, although studies have revealed that the integrity of the BBB is affected during HIV, possible strategies to prevent this have not yet been determined.

Intranasal delivery of antiretroviral drugs has been proposed as a potential strategy to overcome the poor penetration of these drugs into the brain and to target HIV that harbours in the CNS. The intranasal administration of the viral entry inhibitor peptide T has recently been explored for the prevention of neuro-AIDS development such as cognitive impairment associated with HIV. As well as being an entry inhibitor, Peptide T reduces the initial infection of cells expressing CCR5 receptors such as monocytes and microglia and also acts as an antagonist of free gp120 and thereby reduces toxic effects (Hanson and Frey, 2007). More interestingly, peptide T has demonstrated antiviral and immunological benefits in HIV patients receiving intranasal peptide T with decreased viral load in monocyte reservoirs, increased antiviral cytotoxic T-cells with no drug-related toxicity and increased CD4 (Polianova et al., 2003).

A fruitful area of future research will be to determine if there are any systems that can target drug delivery to the brain and also enable the use of recombinant protein therapeutics for this purpose. Novel developments emerging in the field of polymer science and nanotechnology provide an option by which the obstacles of limited brain entry can be surmounted (Kingsley et al., 2006). Although several different polymeric materials have been explored for localised delivery to the brain, these have been unsuccessful due to a variety of factors such as inflammatory responses to implants and their invasiveness. Despite this setback, nanomedicines such as polyethylene glycol-coated liposomes carrying chemotherapeutic drugs for systemic release have been successfully developed and allowed for clinical use, raising the possibility that similar methods for delivery to the brain can be developed in the near future. Other examples of nanomaterials include nanoparticles, polymeric micelles and nanogels (Vinogradov et al., 2004; Kabanov and Gendelman, 2007; Begley, 2004). All have potential as drug delivery systems targeting the brain.A number of recent studies involving polymeric micelles and drug nanosuspensions are of particular relevance to this review. Using brain microvessel endothelial cells Miller et al. (1997) showed that micelles of Pluronic copolymers can affect drug transport by inhibition of P-gp and redirection of vesicular transport (Miller et al., 1997). Subsequently, the same research group found that Pluronic P85 unimers increase accumulation of a P-gp dependent drug in Caco-2 and bovine brain microvessel endothelial cell monolayers by inhibiting P-gp efflux (Batrakova et al., 1998, 2003; Kabanov and Gendelman, 2007). Furthermore, Pluronic P85 inhibited substrate efflux via MRP1 and MRP2 (Miller et al., 1999). Spitzenberger et al. (2007) explored the effects of Pluronic P85, antiretroviral therapy (AZT, 3TC and nelfinavir) or both on a severe combined immunodeficiency animal model of HIVE. This model of HIVE involved inoculating mice with human monocyte-derived macrophages infected with HIV-1 (Spitzenberger et al., 2007). The authors found that Pluronic P85, as well as both the drugs and P85 combined, showed the most significant decrease in percentage of HIV-1 infected monocyte-derived macrophages (8–22% of control) which was superior to the antiretroviral drug group alone (38% of control). This study not only demonstrates that Pluronic P85 increases the penetration of antiretrovirals drugs into the brain but also that block copolymers may have antiretroviral effects, particularly in cells such as macrophages which serve as a viral reservoir in the CNS (Spitzenberger et al., 2007). A recent study has also shown that Pluronic P85 effectively inhibits the interaction of P-gp with the PIs, nelfinavir and saquinavir and that other transporters (including MRP) may also be inhibited by Pluronic P85 (Shaik et al., 2008).Drug nanosuspensions refer to drug particles that are often stabilised by non-ionic PEG-containing surfactants or with mixtures of lipids. This technology is highly advantageous, in the sense that it has a high-drug loading capacity, it is relatively simple to use and can be applied to many drugs. One application of this technology was in studies that used indinavir nanosuspensions for cell-mediated delivery to the CNS. The concept of cell-mediated delivery of nanocarriers to the CNS when loaded with a drug is based upon leukocyte recruitment during an inflammatory response to a pathogen. In particular the process of migration, phagocytosis and exocytosis of inflammatory cells such as macrophages, neutrophils and T cells (Kabanov and Gendelman, 2007). Indinavir nanosuspensions were internalised into bone marrow derived macrophage lysosymes and subsequently the drug was released into the extracellular environment. Thus, these experiments found that indinavir nanosuspension-laden bone marrow derived macrophages were able to carry and release the drug in tissue (Dou et al., 2007). Furthermore, a study in which indinavir nanosuspension bone marrow derived macrophages were administered to HIV-1-challenged mice demonstrated reduced numbers of HIV-infected cells in plasma, lymph nodes, spleen, liver and lungs. The impressive result from this study demonstrates the potential of cell-mediated delivery of nanocarriers to the brain in diseases such as HIV (Kabanov and Gendelman, 2007).It would therefore be advantageous if *in vivo* models could be used to validate novel methods of drug delivery to the brain and if genomic and proteomic techniques can be used to identify new blood–brain and blood–CSF barrier transporters. This could possibly be done if a greater emphasis is placed on the molecular interaction between different transport systems in the BBB, the toxicity induced by drug delivery technologies and molecular imaging of the brain and vasculature (Neuwelt et al., 2008).

The advent of HAART has had a profound effect on HIV/AIDS and its associated complications such as HAD, HIVE and MND. However, to further improve the morbidity associated with this destructive pandemic, methods to circumvent the brain barriers must be accomplished so that sufficient concentrations of anti-HIV drugs can enter the brain. As several studies have acknowledged, this is not an easy task and its difficulty, at least in part, is attributed to the powerful transporters present at both the blood–brain and blood–CSF barriers. Members of the ABC and SLC transporter superfamilies act in concert to protect the brain and consequently restrict the entry of anti-HIV drugs into the brain (Table 3). Several factors add to the complexity of the problem, such as the multi-specificity of the transporters, the diverse expression profiles of transporters in different tissues (e.g. the BBB compared with the choroid plexus), the changing expression of transporters as a consequence of HIV infection and differences between the viral populations in the CNS and the plasma, making treatment immensely difficult. A thorough understanding of each transporter, its location, level of expression and transport potency is needed to help improve the treatment of HIV infection and AIDS.

## Figures and Tables

**Table 1:** Antiretroviral drugs currently approved by the US Food and Drug Administration for the treatment of HIV infection. HAART comprises the combined use of three or more anti-HIV drugs.

NRTIs	NNRTIs	NtRTIs	PIs	Integrase inhibitors	Entry inhibitors	Maturation inhibitors
Zidovudine	Efavirenz	Tenofovir	Saquinavir	Raltegravir	Enfuvirtide^a^	Bevirimat^b^
(AZT)	Nevirapine	(PMPA)	Ritonavir		Maraviroc^c^	
Zalcitabine	Delaviridine		Amprenavir			
(ddC)			Indinavir			
Didanosine			Nelfinavir			
(ddI)			Lopinavir			
Stavudine			Atazanavir			
(d4T)			Fosamprenavir			
Abacavir			Tipranavir			
Emtricitabine			Darunavir			
(FTC)						
Lamivudine						
(3TC)						

Qian et al. (2009) and Kline and Sutliff (2008).

a Fusion inhibitor.

b Currently in clinical trials.

c CCR5 inhibitor.

**Table 2:** A simple scheme to rank CSF-penetrating drugs or the CNS-penetration effectiveness (CPE) ranks, proposed by Letendre et al. (2008a). Rank based on the drug physicochemical characteristics, measured CSF concentrations and CNS efficacy data extracted from publicly available information.

Low	Intermediate	High
Tenofovir (PMPA)	Stavudine (d4T)	Zidovudine (AZT)
Didanosine (ddI)	Lamivudine (3TC)	Abacavir
Zalcitabine (ddC)	Emtricitabine (FTC)	Delavirdine
Nelfinavir	Efavirenz	Nevirapine
Ritonavir	Amprenavir	
Saquinavir	Fosamprenavir	
Enfuvirtide	Atazanavir	
	Indinavir	

**Table 3:** Summary of the current understanding of transporter involvement in anti-HIV drug distribution at blood–CNS interfaces.

Drug name	Drug class	Summary	References
Abacavir	NRTI	P-gp substrate. Inhibits MRP-1, MRP-2 and MRP-3 in a concentration-dependent manner. BCRP substrate^a^	Shaik et al. (2007), Giri et al. (2008), Weiss et al. (2007), Pan et al. (2007)
Zidovudine (AZT)	NRTI	P-gp substrate, MRP-4 and MRP-5 substrate, BCRP substrate.^a^ Removed by a member of the OAT family. OAT1 and OAT3 likely to be responsible for its uptake. OCT possibly involved in uptake. Transported by ENT2. Transported by CNT1 and CNT3	Gollapudi and Gupta (1990), Eilers et al. (2008), Wang and Baba (2005), Wang et al. (2004), Pan et al. (2007), Takasawa et al. (1997), Gibbs and Thomas (2002), Strazielle et al. (2003), Minuesa et al. (2008), Baldwin et al. (2004), Yao et al. (2001), Strazielle et al. (2003)
Nevirapine	NNRTI	Strongly induces the expression and function of P-gp. Inhibits MRP-1, MRP-2 and MRP-3 in a concentration-dependent manner	Stormer et al. (2002), Weiss et al. (2007)
Efavirenz	NNRTI	Induces the expression and function of P-gp. Strongly inhibits MRP-1, MRP-2 and MRP-3 in a concentration-dependent manner	Stormer et al. (2002), Weiss et al. (2007)
Delaviridine	NNRTI	Induces the expression and function of P-gp. Strongly inhibits MRP-1, MRP-2 and MRP-3 in a concentration-dependent manner	Stormer et al. (2002), Weiss et al. (2007)
Ritonavir	PI	P-gp substrate. Increased P-gp activity and expression in a concentration dependent manner. MRP-1 and MRP-2 substrate. Induces the expression of MRP-1 in a concentration-dependent manner. BCRP inhibitor. Possible OCT1 and OCT2 inhibitor	Polli et al. (1999), Perloff et al. (2007), Park and Sinko (2005), Janneh et al. (2005, 2007), Eilers et al. (2008), Gimenez et al. (2004), Gupta et al. (2004), Jung et al. (2008)
Saquinavir	PI	P-gp substrate. MRP-1 and MRP-2 substrate. BCRP inhibitor. Possible OCT1 and OCT2 inhibitor	Kim et al. (1998), Polli et al. (1999), Park and Sinko (2005), Janneh et al. (2005, 2007), Eilers et al. (2008), Gupta et al. (2004), Jung et al. (2008)
Lopanivir	PI	MRP-1 and MRP-2 substrate	Park and Sinko (2005), Janneh et al. (2005, 2007), Eilers et al. (2008)
Emtricitabine	NRTI	Strongly inhibits MRP-1, MRP-2 and MRP-3 in a concentration-dependent manner	Weiss et al. (2007)
Lamivudine (3TC)	NRTI	Inhibits MRP-1, MRP-2 and MRP-3 in a concentration dependent manner. Oatp-2 like transporter has been implicated in its uptake. OCT possibly involved in uptake. OCT1 and OCT2 substrate	Weiss et al. (2007), Gibbs and Thomas (2002), Gibbs et al. (2003a,b), Minuesa et al. (2008), Jung et al. (2008)
Tenofovir (PMPA)	NtRTI	Inhibits MRP-1, MRP-2 and MRP-3 in a concentration dependent manner. OAT1 and OAT3 are high- and low-affinity transporters of PMPA, respectively	Weiss et al. (2007), Cihlar et al. (2001), Izzedine et al. (2005)
Nelfinavir	PI	P-gp substrate. BCRP inhibitor. Possible OCT1 and OCT2 inhibitor	Kim et al. (1998), Gupta et al. (2004), Jung et al. (2008)
Zalcitabine (ddC)	NRTI	OATP has been implicated in its removal. Removed by a member of the OAT family. OAT1 and OAT3 likely to be responsible for its uptake. OCT1 and OCT2 substrate. Transported by ENT2. Transported by CNT3	Gibbs and Thomas (2002), Takasawa et al. (1997), Gibbs and Thomas (2002), Strazielle et al. (2003), Jung et al. (2008), Baldwin et al. (2004), Minuesa et al. (2008)
Didanosine (ddI)	NRTI	Oatp-2 like transporter has been implicated in its uptake. Transported by ENT1 across guinea pig BBB. Transported by ENT2. Transported by CNT1.^b^ Transported by CNT3	Gibbs and Thomas (2002), Gibbs et al. (2003a,b), Baldwin et al. (2004), Li et al. (2001), Minuesa et al. (2008)
Indinavir	PI	P-gp substrate. Possible OCT1 and OCT2 inhibitor.	Kim et al. (1998), Polli et al. (1999), Jung et al. (2008)
Stavudine (d4T)	NRTI	Transported by CNT1^b^	Minuesa et al. (2008)
Amprenavir	PI	P-gp substrate	Polli et al. (1999), Choo et al. (2000)

a Contradictory evidence also exists; see Giri et al. (2008).

b Contradicting evidence also exists; see Minuesa et al. (2008), Chishty et al. (2004), Chang et al. (2004), Cano-Soldado et al. (2004).
