# Corrigendum to “The transport of anti-HIV drugs across blood–CNS interfaces: Summary of current knowledge and recommendations for further research” [Antiviral Res. 82 (2) (2009) A99–A109]

**DOI:** 10.1016/j.antiviral.2009.07.015

**Published:** 2009-11

**Authors:** Lavanya Varatharajan, Sarah A. Thomas

**Affiliations:** a St George's Hospital, Cranmer Terrace, London SW17 ORE, UK; b King's College London, Pharmaceutical Sciences Research Division, Hodgkin Building, Guy's Campus, London Bridge, London SE1 1UL, UK

The authors regret that in the production of Table 2 for this paper, information regarding data for ritonavir boosted drugs was omitted. An amended version of Table 2 is now appearing.

The authors would like to apologise for any inconvenience this may have caused to the authors of this article and the readers of the journal.

## Figures and Tables

**Table 2:** A simple scheme to rank CSF-penetrating drugs or the CNS-penetration effectiveness (CPE) ranks, proposed by Letendre et al. (2008a). Rank based on the drug physicochemical characteristics, measured CSF concentrations and CNS efficacy data extracted from publicly available information. Ritonavir boosted = r.

	Low	Intermediate	High
NRTIs	Tenofovir (PMPA)	Stavudine (d4T)	Zidovudine (AZT)
	Didanosine (ddI)	Lamivudine (3TC)	Abacavir
	Zalcitabine (ddC)	Emtricitabine (FTC)	

NNRTIs		Efavirenz	Delavirdine
			Nevirapine

PIs	Nelfinavir	Amprenavir	Amprenavir-r
	Ritonavir	Indinavir	Indinavir-r
	Saquinavir	Atazanavir	Lopinavir-r
	Saquinavir-r	Atazanavir-r	Fosamprenavir-r
	Tipranavir-r	Fosamprenavir	

Fusion inhibitors	Enfuvirtide		
